# The Multidisciplinary Approach and Surgical Management of GE Junction Adenocarcinoma

**DOI:** 10.3390/cancers16020288

**Published:** 2024-01-09

**Authors:** Meher Oberoi, Md. Sibat Noor, Eihab Abdelfatah

**Affiliations:** Department of Surgery, NYU Langone Health, 120 Mineola Blvd., Suite 320h, Mineola, Long Island, NY 11501, USA; meher.oberoi@nyulangone.org (M.O.); mdsibat.noor@nyulangone.org (M.S.N.)

**Keywords:** gastroesophageal junction cancer, esophagogastric cancer, gastroesophageal cancer, esophageal cancer, gastric cancer, esophagectomy, gastrectomy, proximal gastrectomy

## Abstract

**Simple Summary:**

The management of adenocarcinomas of the gastroesophageal junction is rapidly evolving, making it an interesting and important subject for review. In this review article, we provide a brief overview of the anatomic considerations that impact management and discuss the questions of true biologic nature, incidence, and prognosis that still persist. We discuss the choice of non-operative modalities—chemotherapy, chemoradiation, and immunotherapy—and describe key results from major trials that guide the current standard of care for treatment. We describe in more detail the surgical techniques for esophagectomy, including the advantages and disadvantages of each method, as well as newer surgical techniques for proximal gastrectomy that are especially applicable to Siewert type II tumors.

**Abstract:**

Gastroesophageal (GE) junction adenocarcinoma is an aggressive malignancy of growing incidence and is associated with public health issues such as obesity and GERD. Management has evolved over the last two decades to incorporate a multidisciplinary approach, including endoscopic intervention, neoadjuvant chemotherapy/chemoradiation, and minimally invasive or more limited surgical approaches. Surgical approaches include esophagectomy, total gastrectomy, and, more recently, proximal gastrectomy. This review analyzes the evidence for and applicability of these varied approaches in management, as well as areas of continued controversy and investigation.

## 1. Introduction

Gastroesophageal (GE) junction adenocarcinomas are an aggressive malignancy of growing concern due to their rising incidence in western populations. They are often advanced at the time of diagnosis and have a poor prognosis, with an estimated median 5-year survival of less than 2 years [1,2,3]. Additionally, their association with obesity and Barrett’s esophagus makes them a pertinent public health concern [4,5,6,7]. With several areas of controversy, including their true biologic nature, the anatomic challenges associated with their location at the intersection of two body compartments, and questions of the optimal approach to multidisciplinary and surgical management, GE junction adenocarcinomas are an evolving area of foregut and oncologic surgery.

These cancers are a well-defined but difficult-to-isolate clinical problem, as the current literature often includes them within esophageal or gastric adenocarcinomas. As such, prior reviews have also explained the management of these cancers within the context of gastric or esophageal cancers, when in reality there is significant overlap between the two and the distinction is often arbitrary. Therefore, this review is novel in that it attempts to summarize the plethora of literature to focus on this specific subset of tumors from within the gastric and esophageal cancer literature and define the present state of their multidisciplinary and surgical management as well as future directions.

## 2. Definition of GE Junction Tumors

### Siewert Classification

For the purpose of surgical planning, Siewert’s classification system is the most widely followed for GE junction cancers. Siewert et al. originally defined the GE junction topographically at the angle of His where the esophagus joins the cardia and further divided GE junction tumors into three therapeutically relevant types: Type I tumors are located between 5 and 1 cm proximal to the GE junction; type II tumors are located between 1 cm proximal and 2 cm distal to the GE junction; and type III tumors are located between 2 and 5 cm distal to the GE junction [8,9]. The eighth edition of the AJCC guidelines classifies Siewert type I and II tumors within the staging of esophageal and esophagogastric adenocarcinomas and groups Siewert III tumors with gastric adenocarcinoma [10]. The NCCN guidelines also group treatment of Siewert I and II tumors with esophageal cancer and type III with gastric cancers [11]. However, the biologic origin of the three Siewert-class cancers as gastric or esophageal remains a topic of active research, as will be discussed in more detail later.

**Figure 1:**
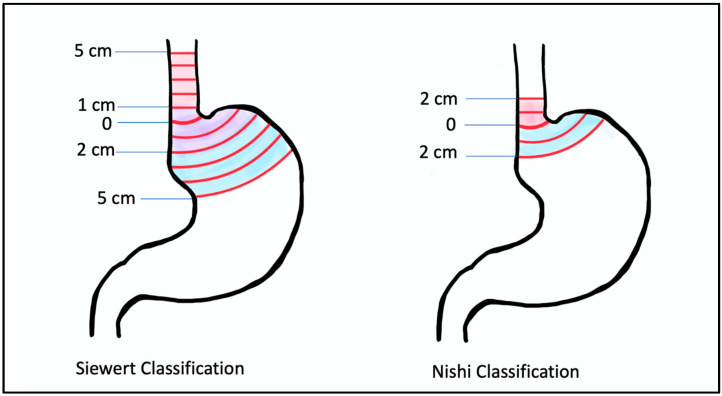
Siewert classification (**left**) and Nishi classification (**right**): Siewert classification describes type I tumors as those that lie between 5 cm and 1 cm from the angle of His; type II tumors as those that lie between 1 cm proximal and 2 cm distal from the angle of His; and type III tumors as those that lie between 2 cm and 5 cm distal to the angle of His. The Nishi Classification System is different in that it limits the definition of the GE junction to 2 cm instead of 5 cm proximal and distal to the angle of His.

While US surgeons fairly uniformly use the Angle of His in concordance with the Siewert classification, there are differences among other specialties in how the GE junction is defined. Gastroenterologists use endoscopic visualization of the proximal extent of the longitudinal gastric folds (different from the squamocolumnar junction or z-line, which lies 3–10 mm proximal to this), and pathologists define it as the most proximal aspect of the gastric folds in an opened surgical specimen. There is no reliable radiographic definition of the GE junction. Additionally, it is important to note that the location of the GE junction may be affected by factors like diaphragmatic movement with respiration, which can cause inter-observer differences, the presence of Barrett’s esophagus, and hiatal hernias, which may change the location of the GE junction [12,13]. Other countries may not use the Siewert system at all. For example, Japan uses the Nishi classification system, which defines GE junction tumors as 2 cm above and below the GE junction (Figure 1).

## 3. Incidence and Prognosis

Between the 1970s and 1990s, the incidence of GE junction adenocarcinoma in the US increased by almost 2.5-fold, followed by a subsequent plateau since the 1990s [2]. This sharp rise in incidence of GE junction cancers garnered concern and raised questions regarding the contributing factors, one of which may have been the change in classification from “non-specific” to “cardia” cancers before the 1990s. A study in 2004 hypothesized that this improved classification had resulted in the perceived sharp increase in incidence. The authors used the SEER database to evaluate the true incidence of cardia cancers in white males from 1974–1998 and reported a decrease in unspecified gastric cancers from 38 to 14%, with a corresponding unadjusted increase in the incidence of cardia cancers of 77%. The adjusted cardia cancer incidence did not see a statistically significant increase, further supporting their hypothesis [14].

Regardless, the most recent estimate of GE junction cancers comes from a 2018 study that used GLOBOCAN to estimate the global disease burden of esophageal and gastric cancers. The authors found a total of 181,000 cases of cardia gastric cancers in 2018 worldwide, with the majority (75%) occurring in Asian countries and 5% occurring in North America. The authors commented on the increasing incidence in younger populations in otherwise low-incidence countries such as the US and UK [3]. This rising incidence may be attributable to the increasing prevalence of obesity, GERD, and Barrett’s esophagus in the Western world. While *H. pylori* remains a notable risk factor in distal gastric cancers in Asian countries, it is not associated with GEJ junction cancers in western populations [2,15].

Between 1973 and 2008, the overall 5-year survival of patients with GE junction adenocarcinoma improved from 8–12% to 17%. This improvement was attributed to improved diagnosis and treatment. However, neoadjuvant treatment before surgery, which is now considered a standard of care, was not widely used until at least 2012. Neoadjuvant chemotherapy and/or chemoradiation and downstaging of tumors have resulted in increased rates of achieving R0 resection and improved prognosis, with 10-year survival estimates of 36% in randomized trials [16,17]. However, the question of overall prognosis in the era of neoadjuvant therapy at a population level remains unanswered. Interestingly, Nakauchi et al. have developed a nomogram to help predict disease-specific survival in patients with GE junction tumors who receive neoadjuvant treatment, which may prove to be a useful prognostic tool at the individual level [18]. Predictors of improved disease-specific survival in their model were 30 or more negative nodes, 30% or greater pathologic response, and 90% or greater pathologic response.

## 4. Biological Characteristics of GE Junction Cancers

The traditional thinking in regard to the origin of GE junction adenocarcinomas has been that Siewert type I adenocarcinomas, or those just proximal to the GE junction, are esophageal in origin, arising from intestinal metaplasia of the squamous esophageal epithelium, or Barrett’s esophagus. This contradicts the initial description of Barrett’s esophagus as a proximal migration of the stomach epithelium [19]. Cancers at or just distal to the GE junction are thought to be gastric in origin. However, more recently, the very origin of Barrett’s esophagus has come into question. In a 2012 mouse model study by Quante et al., the authors found that both Barrett’s esophagus and esophageal adenocarcinoma arose from gastric progenitors [20]. Subsequently, in a 2017 analysis by The Cancer Genome Atlas Research Network, comprehensive molecular genomic profiling found a strong resemblance between esophageal adenocarcinomas and the chromosomally unstable variant of gastric cancer, further supporting the hypothesis of a common gastric origin for all GE junction adenocarcinomas [21]. Several studies have since provided additional compelling evidence to support a gastric origin for all GE junction adenocarcinomas [22,23,24,25].

Despite the evidence suggesting a common gastric origin, there are notable differences in the cancers of the GE junction and those located more distally in the stomach. An association with microsatellite instability, Ebstein-Barr virus infection, *Helicobacter pylori* infections, and diffuse-type tumor pathology are all more common in non-GE junction or distal gastric cancers, in contrast to a stronger association with obesity, GERD, and Barrett’s esophagus in GE junction cancers [2,20].

## 5. Early-Stage Disease

Early-stage cancers of the GE junctions, which are limited to the mucosa, well differentiated, small (<2 cm), and without nodal involvement, lymphovascular invasion, or perineural invasion, do not require systemic therapy and are treated with upfront resection. This is due to the low nodal metastatic rate of T1a lesions [26]. This may be achieved via surgical excision or, more preferably, endoscopically, if feasible, via techniques such as endoscopic mucosal resection (EMR) or endoscopic submucosal dissection (ESD), often in conjunction with ablation [11,27].

## 6. Resectable Locoregional and Locally Advanced Disease

### 6.1. Chemoradiation and Chemotherapy

Chemoradiation has been used in the multidisciplinary treatment paradigm of GE junction adenocarcinoma for the past two decades. The CROSS trial, published in 2012, was a landmark study that compared chemoradiation followed by surgery versus surgery alone in esophageal and GE junction cancer and found improved 5-year overall survival with the addition of chemoradiation. However, the degree of benefit was greater in the squamous cell carcinoma group, with pathologic complete response rates of 49% in this group compared to 23% in the adenocarcinoma group, which included GE junction tumors. For patients with adenocarcinoma, median overall survival was 43.2 months in the neoadjuvant chemoradiotherapy plus surgery group and 27.1 months in the surgery alone group (*p* = 0.038) [16,28]. Based on this study, neoadjuvant chemoradiation became the standard of care for patients with esophageal and GE junction cancers.

However, concomitant with these results, the MAGIC trial published its findings in 2006 demonstrating significant survival benefit with a joint pre(three cycles)- and post(three cycles)- operative chemotherapy regimen of Cisplatin, Fluorouracil, and Epirubicin (ECF) when compared with surgery alone; 5-year overall survival was 36% in the perioperative chemo group vs. 23% in the surgery alone group, *p* = 0.009 [29]. The MAGIC trial included only adenocarcinomas, 11.2% of which were classified as esophagogastric junction tumors, as well as 14.8% lower esophageal and 74% gastric tumors, and it is unclear how many of those gastric or lower esophageal tumors would have fallen into the Siewert classification of GE junction tumors. Nevertheless, there was a clear survival benefit with chemotherapy alone in a group that, unlike the CROSS trial, consisted of exclusively adenocarcinomas without squamous cell carcinoma. This highlights the importance of the different biologic behaviors of squamous cells vs. adenocarcinomas and the differences in response to therapy. The MAGIC trial was followed by the FLOT trial in 2019, which included gastric or GE junction adenocarcinomas and compared the MAGIC trial regimen to perioperative Docetaxel, Oxaliplatin, Leukovorin, and 5-FU. They reported significantly improved median survival with the FLOT regimen (50 months vs. 35 months; *p* = 0.012) and improved 5-year overall survival (45% vs. 36%) [30].

The MAGIC trial did not specifically comment on rates of R0 resection but did report tumor shrinkage in the chemotherapy group (3 cm vs. 5 cm, *p* < 0.001), downstaging with a higher proportion of T1-T2 tumors (51.7% vs. 36.8%, *p* = 0.002), and less advanced nodal disease (84.4% vs. 70.5%, *p* = 0.01) in the chemotherapy group [29]. The CROSS trial found higher rates of R0 resection in the chemoradiation group when compared with upfront surgery (92% vs. 69%, *p* < 0.001) [31]. Both trials found no difference in the rates of postoperative complications and 30-day mortality when compared with surgery alone. The FLOT trial reported higher rates of R0 resection in the FLOT group (85% vs. 778%, *p* = 0.0162) and also found no difference in postoperative complications or 30-day mortality when comparing the FLOT regimen with ECF/ECX [30].

Because both the neoadjuvant chemoradiation and perioperative chemotherapy trials included GE junction tumors, this raised the management challenge of whether to treat GE junction adenocarcinoma with neoadjuvant chemoradiation or chemotherapy. The POET trial compared neoadjuvant chemotherapy followed by surgery versus neoadjuvant induction chemotherapy followed by chemoradiation, then surgery. There were higher rates of pathologic complete response and node-negative resection in the chemoradiation group. Local progression-free survival at 5 years after resection was significantly improved by chemoradiotherapy (*p* = 0.01), but there was no significant difference in overall survival [32,33]. A study by Klevebro et al. analyzing 181 patients showed similar findings with the addition of radiotherapy to neoadjuvant chemotherapy, resulting in a higher histological complete response rate, a higher R0 resection rate, and a lower frequency of lymph node metastases but without a significant change in overall survival, with a three-year overall survival of 49% vs. 47% (*p* = 0.77) [34]. Finally, the NeoResI trial in 2019 reiterated these findings by comparing neoadjuvant chemotherapy to neoadjuvant chemoradiotherapy in esophageal and GE junction cancers and found no difference in 5-year progression-free or overall survival between the two groups [35].

Therefore, the results of these trials suggest that while the addition of chemoradiation may improve local factors such as pathologic complete response, node negative rates, and rates of R0 resection, overall survival rates are not significantly improved. GE junction adenocarcinomas primarily recur with distant metastatic and not locoregional recurrence, which may be why a more aggressive locoregional control approach such as chemoradiation does not result in improved overall survival. The difficulty in decisively drawing a conclusion on chemoradiation vs. chemotherapy in GE junction adenocarcinoma arises from the fact that they are included in trials of both esophageal and gastric cancers, as well as trials that include squamous cell carcinomas, which have a different biologic behavior and response to chemoradiation. The ongoing ESOPEC trial randomized esophageal and GE junction adenocarcinoma to neoadjuvant chemoradiation with the CROSS protocol vs. perioperative chemotherapy with the FLOT protocol and will hopefully give the definitive answer to this management challenge [36].

### 6.2. Immunotherapy

Immunotherapy has been incorporated into the management of GE junction adenocarcinoma. The CheckMate 577 trial randomized patients with stage II or III esophageal or GE junction cancer who had undergone chemoradiation followed by R0 resection and had residual pathological disease on the resection specimen to receive adjuvant PD-1 checkpoint inhibition with nivolumab vs. placebo. They found that patients who received nivolumab had a median disease-free survival of 22.4 months, compared to 11.0 months (*p* < 0.001). The study concluded that the risk of distant recurrence or death was 26% lower with nivolumab than with placebo [37]. Based on this, NCCN guidelines now recommend adjuvant immunotherapy for resected esophageal or GE junction cancers that received neoadjuvant chemoradiation and have residual pathological disease on final pathology.

There is limited information on the impact of neoadjuvant immunotherapy in relation to the short-term outcomes of surgery for GE junction cancer. Early results from the NEONPIGA trial (evaluating MMR deficient/MSI-high gastric and GE junction adenocarcinoma receiving neoadjuvant nivolumab and iplimumab followed by surgery plus adjuvant nivolumab) have shown complete pathologic response in 17/29 patients and complete R0 resection in 29/29 patients at a median follow-up of 14.9 months. Six of twenty-nine patients suffered grade III or higher postoperative complications, and one death occurred within 3 days of surgery secondary to cardiopulmonary causes in this group [38].

## 7. Tailored Therapy for HER-2-Positive GE Junction Adenocarcinoma

There is promising data for the use of trastuzumab in HER2-positive gastric and GE junction cancers. The ToGa trial reported a significant survival advantage (13.8 months vs. 11.1 months, *p* = 0.0046) with the addition of Traztuzumab to the capecitabine/cisplatin/FU regimen in patients with HER-2-positive gastric and GE junction cancers [39]. The HER-FLOT trial evaluated patients with HER2-positive gastric and GE junction cancers staged cT2 or higher and/or cN+ without distant metastases and reported a pathologic complete response rate of 21.4% in patients who received trastuzumab in addition to the FLOT regimen [40]. The reported safety concerns with the regimen. However, Phase III RTOG 1010, which compared CROSS vs. CROSS + Trastuzumab, found no additional benefit in pathologic response or disease-free survival [41]. Thus far, this suggests that there is an advantage when adding Trastuzumab to chemotherapy but not when added to chemoradiation; however, more long-term data are needed.

## 8. Surgery

Due to the anatomic challenge of GE junction tumors being located at the interface of the thoracic and abdominal cavity, the approaches to surgical management are not well defined and can be variable based on institution and surgeon preference. Typically, Siewert type I tumors have been managed with esophagectomy and Siewert type III with a total gastrectomy, with Siewert II demonstrating the greatest degree of variability. This section reviews the different surgical approaches and outcomes (Table 1).

### 8.1. Transhiatal Esophagectomy

This operation utilizes abdominal incisions and a neck incision for a transcervical anastomosis. The stomach is mobilized, and the gastric conduit is created while preserving the right gastroepiploic artery. The left gastric artery and vein are identified and divided at their origin to perform a lymphadenectomy. A penrose drain is placed around the GE junction for caudal retraction of the stomach and lower esophagus. The nodal tissue in the lower mediastinum is then dissected under direct visualization. The distal 5–10 cm of mediastinal esophagus is also dissected first with an electrosurgical device and then bluntly with tactile guidance from a bougie placed in the esophagus if performed open. Using minimally invasive or robotic approaches can often achieve visualization higher up into the mediastinum. In the neck, an incision is created, dividing the omohyoid muscle while identifying and preserving the left recurrent laryngeal nerve. The dissection is carried down to the prevertebral fascia and esophagus, until the surgeon is able to pass the index finger between the prevertebral fascia and esophagus and subsequently dissect the cervical esophagus off the trachea. A penrose drain is placed around the esophagus here, and the esophagus is further mobilized. It is then delivered through the neck and divided. The gastric conduit is then drawn up and out of the cervical incision, delivering the stomach into the neck. The esophagus may be trimmed further, and a handsewn or stapled esophagogastric anastomosis is created [42,43].

### 8.2. Ivor–Lewis Esophagectomy

This operation utilizes abdominal and thoracic incisions for a transthoracic anastomosis. The stomach is mobilized while preserving the right gastroepiploic artery. The left gastric pedicle is divided at its origin. The phrenoesophageal membrane is divided, and the right crus may be divided if necessary as well. The conduit is created by dividing the stomach along the lesser curvature, starting proximally to the right gastric artery and ending in between the cardia and the fundus. A pyloric drainage procedure may be performed, such as a pyloromyotomy, pyloroplasty, or botox injection; however, recent studies have questioned the utility of this [44,45].The patient is repositioned, and through a thoracic incision, the thoracic esophagus is mobilized and transected, and a lymphadenectomy of the paraoesophageal, inferior pulmonary ligament, and subcarinal lymph nodes is performed. The conduit is then pulled into the chest, and an esophagogastric anastomosis is performed. Common techniques for anastomosis include stapling with an EEA stapler, side-to-side stapled anastomosis with a linear stapler, or handsewn anastomosis. A pleural flap, omental flap, or intercostal muscle flap is used for coverage of anastomosis [43,46].

### 8.3. McKeown Esophagectomy

This operation combines the Ivor–Lewis and transhiatal approaches and utilizes abdominal, thoracic, and neck incisions. The thoracic portion of the operation is completed first in the left lateral decubitus position. The patient is then repositioned to the supine position for the creation of the gastric conduit, and the anastomosis is performed in the neck [47].

### 8.4. Minimally Invasive Approaches

Esophagectomies are increasingly being performed with minimally invasive approaches, either robotically or laparoscopically/thoracoscopically. The TIME trial was a multicenter, randomized controlled trial that showed significantly lower pulmonary infection rates (12% vs. 34%, *p* = 0.005), decreased length of stay, decreased postoperative pain, and improved quality of life at 6 weeks with MIE compared to open esophagectomy [48]. MIE was performed via laparoscopy and thoracoscopy with the patient in the prone position. Another randomized controlled trial compared open transthoracic esophagectomy to robot-assisted thoracolaparoscopic esophagectomy in 112 patients from 2012 to 2016. Robotic esophagectomy was associated with significantly fewer surgery-related and cardiopulmonary complications, less blood loss, lower postoperative pain, an improved quality of life score after discharge, and improved functional recovery at 2 weeks, with no difference in short- or long-term oncologic outcomes up to 40 months [49]. The authors noted comparable lymph node retrieval and disease-free survival (24 months vs. 26 months). Several studies have pointed to higher lymph node retrieval with minimally invasive approaches, though this is not reported consistently [50,51,52,53], and multiple studies have reported equivalent overall survival at 5 years with minimally invasive approaches [51,54].

### 8.5. Ivor–Lewis vs. Transhiatal Esophagectomy

The advantages of the Ivor–Lewis approach result from improved transthoracic visualization, which allows for extensive lymph node dissection and a higher lymph node yield [55]. While older studies reported higher rates of pulmonary complications with transthoracic approaches [56], recent studies that are more representative of the modern era with minimally invasive surgery and improved perioperative care have shown results varying from no difference to actually fewer pulmonary complications with transthoracic esophagectomy when compared with transhiatal approaches [55,57]. Another advantage of the transthoracic approach is the lower anastomotic leak rate [58]. However, though the leak rate is lower, the potential consequences of a leak in the chest are worse than those of a leak in the neck, which may be easier to manage with debridement and wound care. The transhiatal operation avoids thoracic incisions but also limits visualization and is associated with lower lymph node yield [55]. Additionally, the cervical anastomosis in the transhiatal approach is associated with a risk of recurrent laryngeal nerve injury and swallowing dysfunction [59]. Importantly, while neither approach is established as oncologically superior for the GE junction, a few studies have now demonstrated survival benefits with transthoracic esophagectomy when compared with transhiatal approaches overall [55,60,61].

### 8.6. Technique for Total Gastrectomy

Total gastrectomy is often performed for Siewert III and possibly Siewert II tumors. In this technique, the greater omentum is separated from the transverse mesocolon, the lesser sac is entered, and the plane is extended proximally and distally. A complete omentectomy may be performed. The right gastroepiploic artery is then identified and divided at its origin from the gastroduodenal artery. The infrapyloric nodal tissue is mobilized with the specimen. The greater curvature of the stomach is further dissected with careful division of the short gastric vessels up to the phrenoesophageal ligament. The lesser curvature is then mobilized. The right gastric artery is identified and divided at its origin from the common hepatic artery. The duodenum is then circumferentially dissected distal to the pylorus and divided. The specimen is reflected anteriorly, and the left gastric artery is identified and divided, taking all nodal tissue. The distal esophagus is mobilized and divided, and reconstruction is performed with a Roux-en-Y esophagojejunostomy [43]. NCCN guidelines recommend the removal of at least 16 lymph nodes with a complete D2 lymph node dissection when performing total gastrectomy for gastric cancer [62]. This involves the removal of perigastric nodal tissue as well as nodal tissue along the named celiac axis vessels, i.e., the left gastric, common hepatic, celiac, and splenic arteries. D2 lymphadenectomy has been shown to have a survival benefit in Eastern studies, but these results have not been replicated in Western trials [63]. Multiple studies consistently report a shorter length of stay, less blood loss, and fewer wound complications with minimally invasive total gastrectomy. Rates of other perioperative complications and overall survival are comparable with the two approaches [64,65,66,67,68,69,70,71,72].

### 8.7. Esophagectomy versus Total Gastrectomy

In Siewert II tumors, clinical practice is variable between esophagectomy, gastrectomy, and, more recently, proximal gastrectomy. Regardless of the approach, an R0 resection is considered an independent predictor of long-term survival [9]. Additionally, a few studies have noted improved survival with a wider margin [73,74]. With this in mind, total gastrectomy poses certain challenges as it is difficult to achieve a proximal margin of more than 3–4 cm on the esophagus. A few studies have reported higher rates of positive margins with gastrectomy when compared with esophagectomy [75,76,77] and higher lymph node retrieval with esophagectomy. In one registry-based analysis by Kamarajah et al., the authors reported comparable lymph node harvest but improved overall survival with esophagectomy [78]. However, these reports are not consistent and have not translated reliably into improved overall survival [75,77,79,80].

When compared with esophagectomy, gastrectomy has been associated with some improved quality of life parameters, with fewer patients reporting reflux, swallowing dysfunction, and cough [81,82,83]. Esophagectomy, especially transthoracic esophagectomy, has been reported to have higher rates of pulmonary and cardiac complications [84], while gastrectomy is associated with an increased risk of dumping syndrome [83]. At present, there are no randomized controlled studies supporting esophagectomy over gastrectomy or vice versa for Siewert II GE junction adenocarcinoma. The CARDIA trial is an ongoing multicenter randomized clinical trial comparing transthoracic esophagectomy and transhiatal extended gastrectomy, which will provide greater insight [85].

## 9. Proximal Gastrectomy

### 9.1. Rationale

As discussed above, there is variability in the surgical approach chosen for Siewert 2 and 3 GE junction adenocarcinomas, especially Siewert 2. For tumors in the same location, some surgeons will choose to perform esophagectomy, while others choose to perform total gastrectomy (TG). These have traditionally been seen as the two surgical options; however, there is increased interest in and utilization of proximal gastrectomy (PG) as an additional option.

Tumors of the distal stomach are treated with subtotal distal gastrectomy, where the distal 50–60% of the stomach is resected along with appropriate lymphadenectomy and an anastomosis created to the proximal stomach. This is preferred to total gastrectomy due to less weight loss and better functional outcomes with equivalent oncologic outcomes [86,87,88]. On the other hand, proximal gastrectomy for proximal gastric or GE junction tumors has not been adopted in the same way due to concerns about oncologic resection margins, the adequacy of lymphadenectomy, and reconstruction challenges. Recent studies have evaluated these concerns, and proximal gastrectomy is increasingly being utilized as an alternative to total gastrectomy in Siewert 2 and 3 GE junction cancers.

### 9.2. Oncologic Concerns

The first and most important question is whether PG is an oncologically equivalent approach compared to TG for type 2/3 GE junction cancers. In regard to the extent of lymphadenectomy during resection, one study has quantified positivity rates across each lymph node station. A prospective nationwide Japanese study assessed rates of lymph node metastases at abdominal and mediastinal lymph node stations for cancers at the GE junction to determine rates of nodal metastatic disease at each lymph node station [89]. For adenocarcinoma, the lymph node stations with the highest rates of nodal positivity were the right pericardial (36.4%), left pericardial (27.5%), lesser curvature (38.8%), and left gastric (23.2%). Celiac and proximal splenic nodes also had rates of positivity above 10%. However, all other abdominal nodal stations had rates below 10%, with nodes along the distal stomach, distal greater curvature, and pylorus having rates of under 2%. Additionally, for tumors with 1–2 cm of esophageal involvement, rates of lower mediastinal paraesophageal node positivity were 6.4%, and in tumors with less than 1 cm of esophageal involvement, the rate of nodal positivity was 0.9%. Therefore, the nodal stations most likely to be involved in GE junction adenocarcinomas with <2 cm of esophageal involvement would be adequately cleared with PG without requiring further esophagectomy.

Several studies have supported the oncologic equivalence of PG to TG for GE junction and proximal gastric adenocarcinoma. A retrospective single-center study out of Korea compared outcomes between 50 laparoscopic PG and 81 laparoscopic TG for cT1-T2N0M0 adenocarcinoma in the upper 1/3 of the stomach and found no difference in rates of overall survival [90]. A more recent single institution study out of China retrospectively compared PG with double tract reconstruction (PGDT) to TG in cT1-T2N0M0 Siewert 3 GE junction adenocarcinoma and found no difference in recurrence-free or overall survival rates between the two groups in pathologic stage 1, 2, or 3 disease [91]. Similarly, a retrospective study in clinical stage 1 proximal gastric cancer [92] and a retrospective study in early stage Siewert 2/3 GE junction adenocarcinoma [93] showed similar findings of equivalent overall survival between PG and TG.

A retrospective, multicenter Italian study did not limit selection criteria to only early-stage gastric adenocarcinoma and included 457 patients, over 30% with stage 2 and 25% with pathologic stage 3 disease [94]. Despite not being limited to only early-stage cancers, the results were similar, with no significant difference in overall survival between PG and TG. In fact, there was a trend towards improved survival in the PG group (5-year OS: 56.7% PG vs. 46.5% TG, *p* = 0.07). The 2021 edition of the Japanese gastric cancer treatment guidelines recommends proximal gastrectomy in GE junction tumors and proximal tumors where at least half of the distal stomach can be preserved; however, it limits this to early-stage cT1N0 tumors [88]. An international, multicenter prospective cohort study is currently underway to compare minimally invasive PG to TG in proximal gastric and GE junction adenocarcinoma in terms of oncologic and functional/quality-of-life outcomes [95]. Patients will not be limited to only stage 1 or early-stage tumors, and patients receiving neoadjuvant therapy are not excluded. Therefore, it will shed more light on the applicability of PG to patients with more advanced disease and those receiving neoadjuvant therapy.

### 9.3. PG Reconstruction Techniques and Outcomes

While these studies have supported the rationale that PG is at least oncologically equivalent to TG, another concern is functional outcomes and complications. TG has been associated with greater postoperative weight loss as a percentage of body weight (15–20%) than distal subtotal gastrectomy (5–10%) [87]. This is important not only for quality of life but also oncologic outcomes, as studies have shown that greater postoperative weight loss is associated with decreased compliance with adjuvant chemotherapy [96] and early recurrence [97]. Therefore, if PG can be similarly shown to decrease postoperative weight loss and improve quality of life for GE junction cancers compared to TG, then it would support its use in appropriately selected patients.

### 9.4. Esophagogastric Anastomosis

PG with direct anastomosis between the distal stomach and esophagus (esophagogastrostomy) is the fastest and most straightforward anastomotic technique, with preservation of the normal route of enteral contents through the esophagus, stomach, and into the duodenum. However, the initial experience with this technique had significant functional limitations. PG with esophagogastrostomy is associated with high rates of severe gastroesophageal reflux and resultant reflux esophagitis [98,99,100,101] due to the loss of the lower esophageal sphincter, impaired gastric emptying function of the distal stomach, and preservation of the pylorus. A systematic review of reconstruction methods found a 28.6% rate of reflux esophagitis (ranging between 20% and 65.2%), as well as a 21.8% rate of residual food and a 15.4% rate of stenosis [102].

Modifications to the esophagogastric anastomosis have been performed to attempt to reduce these complications, with limited success. Tabularization of the gastric tube has been reported in several retrospective studies with small sample sizes, with reported rates of reflux esophagitis of 5.7% [103] and 26.7% [104]. Another technique is the creation of a fundoplication wrap as an anti-reflux mechanism at the time of esophagogastric anastomosis, with rates of reflux esophagitis of 22–30% in retrospective series [105,106]. The study by Nakamura et al. did report a significant decrease in reflux esophagitis to 3.6% in patients who received a fundoplication wrap of ≥180 degrees; however, stricture rates were high for both groups at 22%. Finally, a study by Kuroda et al. reported on a novel “double flap” reconstruction technique in which a seromuscular flap is created from the stomach wall through which the esophageal lumen is hand-sewn to the stomach lumen, and then the seromuscular flap is closed on top of the area of the esophagogastric anastomosis [107]. Reflux esophagitis rates were not reported in this study. Based on the results of these studies, a consistent and reliable method to reduce the rate of reflux esophagitis and stricture formation in esophagogastric anastomosis after PG has not been established.

### 9.5. Jejunal Interposition and Double Tract Reconstruction

Alternative reconstructions of the esophagogastric anastomosis have been developed. The two most commonly utilized and reported are jejunal interposition and double-tract reconstruction. Both of these reconstructions utilize the jejunum as a conduit between the esophagus and distal gastric remnant.

Jejunal interposition (JI), first described in 1955 by Merendino et al., involves transecting the proximal jejunum about 20–30 cm distal to the ligament of Treitz while maintaining its mesentery and vascular supply. An esophagojejunal anastomosis is then performed, and 10–15 cm distally, a gastrojejunal anastomosis is performed. The jejunum distal to the gastrojejunal anastomosis is transected, and a jejunojejunal anastomosis is then performed to restore continuity of the GI tract [108,109].

In double-tract reconstruction (DTR), a Roux-en-Y esophagojejunostomy is performed, similar to a total gastrectomy. However, an additional side-to-side anastomosis is created between the roux limb and the distal gastric remnant, which allows for the passage of food contents either into the gastric remnant or down the roux limb to the jejunojejunal anastomosis [109].

The outcomes of these JI and DTR reconstructions have been shown to significantly reduce or eliminate the issues of reflux esophagitis and anastomotic stricture formation seen with esophagogastric reconstruction techniques [100,102,109,110]. Residual food rates of 8.5%–48.9% have been reported with these techniques, possibly related to delayed gastric emptying of the distal stomach remnant [100,102]. However, proponents of DTR argue that residual food identified endoscopically is asymptomatic in the majority of patients and less of a concern with this reconstruction due to the alternative route of food passage down the efferent jejunal limb from the gastrojejunostomy anastomosis [111].

JI and DTR avoid issues of reflux esophagitis and anastomotic stricture but may have high rates of residual food, possibly related to DGE. However, with DTR, even if DGE is present, there is an alternative route of food down the jejunum.

### 9.6. Comparison of PG to TG Outcomes

Multiple studies have compared functional, nutritional, and quality of life outcomes between PG and TG. A randomized controlled trial comparing JI, DTR, and TG found decreased reflux esophagitis rates and improved nutritional parameters in the JI and DTR groups compared to TG in terms of hemoglobin, serum protein, serum albumin, vitamin B12, and weight loss up to 18 months postoperatively [109]. A 10-year propensity-matched study had similar findings of less weight loss and preservation of more skeletal muscle, measured by fat-free mass index, up to 2 years postoperatively in the PG group compared to the TG group [91]. A randomized controlled trial evaluating specifically hemoglobin level and vitamin B12 supplementation between laparoscopic PG-DTR and laparoscopic TG also found lower hemoglobin change and less B12 supplementation in the PG-DTR group, with better quality of life scores in physical and social functioning [112]. Quality of life scores in nausea/vomiting and weight loss were not found to be significantly different between the two groups.

A 2019 meta-analysis comparing PG-DTR and TG evaluated anastomotic stricture rates, reflux esophagitis, and vitamin B12 supplementation and found no difference between the groups in esophagitis or stricture rates but a significantly lower B12 supplementation requirement favoring PG-DTR [113]. A 2023 systematic review and meta-analysis by Hipp et al. compared PG-DTR and TG and found significantly lower reductions of hemoglobin, less B12 supplementation, and less weight loss with PG-DTR [114]. Finally, the PGSAS-45 was a multi-institutional Japanese study that evaluated patient-reported outcomes in comparing PG and TG. They found that changes in body weight, necessity for additional meals, ability to work, and dissatisfaction with working were significantly better in the PG group than in the TG group [115].

## 10. Future Directions

The treatment of GE junction cancers is evolving, and future studies will further define the best approaches from a multidisciplinary management and surgical management standpoint. The question of superiority between chemotherapy and chemoradiation in GE junction adenocarcinoma will be addressed by the results of the ongoing ESOPEC trial [36]. Further trials evaluating the role of immunotherapy neoadjuvantly, especially in mismatch-repair-deficient and PD-L1-high tumors, will also shed further light, as will the additional use of targeted therapeutics such as HER-2 inhibitors [38,116]. Finally, surgical management will continue to evolve with the increased integration of robotics platforms and additional trials evaluating proximal vs. total gastrectomy, such as the international multi-center trial that is currently ongoing [95].

## 11. Conclusions

GE junction adenocarcinoma is a growing public health concern around the world, with a direct correlation to increasing rates of obesity and GERD. The anatomic location and biological characteristics of these tumors present unique management challenges. Advancements have been made with the incorporation of neoadjuvant therapy and improvements in surgical techniques, including minimally invasive surgery. Future research should include defining the biology of these tumors, evaluating the use of chemotherapy vs. chemoradiation, performing fewer radical operations using proximal gastrectomy, and incorporating targeted molecular therapy and immunotherapy, which will allow us to treat these tumors with greater efficacy.

## Figures and Tables

**Table 1:** Options for surgical management of Sierwert type II and III GE junction adenocarcinomas.

Technique	Clinical Indication	Advantages/Disadvantages	
Esophagectomy			
	• Esophageal extension >2 cm	Pro:	Complete mediastinal lymphadenectomy
	• Mediastinal lymphadenopathy		Maintenance of gastric antrum and physiologic food passage
		Con:	Requires thoracic or mediastinal dissection
			Intrathoracic or neck anastomosis with associated complications
			Gastric emptying issues
Total gastrectomy			
	• <2 cm esophageal involvement	Pro:	Complete intra-abdominal lymphadenectomy
	• Diffuse gastric involvement		Complete resection of all gastric mucosa, no concern for gastric margin or recurrence
	• Advanced stage disesase (T3–T4 or N1)		
		Con:	Profound weight loss, 15-20% of body weight
			Diminished QOL compaired to partial gastrectomy
			Anemia due to loss of antrum, B12 supplementation required
Proximal gastrectomy			
	• <2 cm esophageal involvement	Pro:	Less weight loss than total gastrectomy
	• Early stage, localized disease (T1–2, N0)		Improved quality of life outcomes
	• May be appropriate for advanced stage disease, still an active area of investigation		Maintenance of antrum
		Con:	Concern about gastric margin or adequacy of lymphadenectomy in advanced stage disease
			Gastric emptying issues
			Reflux esophagitis in esophagogastrostomy
Method			
Esophagogastrostomy		Pro:	Simplest, most efficient reconstruction
		Con:	High rates reflux esophagitis, anastomotic stenosis and food retention
Jejunal Interposition		Pro:	Decreased reflux esophagitis and anastomotic stenosis
		Con:	Increased operative time, multiple anastomoses, residual food
Double-Tract Reconstruction		Pro:	Decreased reflux esophagitis, alternative route for any residual food
		Con:	Increased operative time, multiple anastomoses
